# Biosecurity risks posed by a large sea-going passenger vessel: challenges of terrestrial arthropod species detection and eradication

**DOI:** 10.1038/s41598-019-55554-4

**Published:** 2019-12-18

**Authors:** Simon J. McKirdy, Simon O’Connor, Melissa L. Thomas, Kristin L. Horton, Angus Williams, Darryl Hardie, Grey T. Coupland, Johann van der Merwe

**Affiliations:** 1 Harry Butler Institute, Murdoch University, Murdoch, WA 6150 Australia; 2 Chevron Australia, 256 St Georges Tce, Perth, WA 6000 Australia; 3 Department of Primary Industries and Regional Development Western Australia, Division of Agriculture and Food, 4 Baron-Hay Court, South Perth, 6151 Australia

**Keywords:** Invasive species, Biodiversity

## Abstract

Large sea-going passenger vessels can pose a high biosecurity risk. The risk posed by marine species is well documented, but rarely the risk posed by terrestrial arthropods. We conducted the longest running, most extensive monitoring program of terrestrial arthropods undertaken on board a passenger vessel. Surveillance was conducted over a 19-month period on a large passenger (cruise) vessel that originated in the Baltic Sea (Estonia). The vessel was used as an accommodation facility to house workers at Barrow Island (Australia) for 15 months, during which 73,061 terrestrial arthropods (222 species - four non-indigenous (NIS) to Australia) were collected and identified on board. Detection of *Tribolium destructor* Uytt., a high-risk NIS to Australia, triggered an eradication effort on the vessel. This effort totalled more than 13,700 human hours and included strict biosecurity protocols to ensure that this and other non-indigenous species (NIS) were not spread from the vessel to Barrow Island or mainland Australia. Our data demonstrate that despite the difficulties of biosecurity on large vessels, stringent protocols can stop NIS spreading from vessels, even where vessel-wide eradication is not possible. We highlight the difficulties associated with detecting and eradicating NIS on large vessels and provide the first detailed list of species that inhabit a vessel of this kind.

## Introduction

Managing the pathways by which non-indigenous species (NIS) are introduced and spread is considered the most effective method for preventing species’ invasions^1–3^. Cargo and passenger vessels are considered a major pathway for the spread of NIS between countries^2,4–6^. Research on the biosecurity risk posed by large vessels focusses primarily on invasive marine species, with emphasis on the mechanisms designed to reduce introduction and spread of marine pests e.g.^7,8^. Marine vessels also provide a vector for terrestrial NIS species, e.g.^3,9,10^, however only a few studies focus on terrestrial NIS, and most of these are on well-known invasive terrestrial vertebrates, such as *Rattus* species^11^.

There is good evidence that terrestrial arthropods use marine vessels for transoceanic travel, e.g.^3,12^, and establishment in new areas, e.g.^13^. However, only two studies to date have provided a detailed assessment of the diversity of terrestrial arthropods occupying large vessels (see^14,15^). The arthropods detected include cockroaches (Blattoidea), moths (Lepidoptera), flies and mosquitoes (Diptera), wasps and ants (Hymenoptera) and beetles (Coleoptera)^14,15^. Understanding the terrestrial arthropod fauna that may be present on vessels is essential for assessing the biosecurity risk these vessels pose and to ensure vessel NIS management programmes are successful. This is particularly pertinent, given that border inspection has been described as ineffective in detecting a high percentage of non-indigenous incursions^16^.

Passenger vessels, particularly cruise vessels, have the potential to pose a risk to high value marine and terrestrial ecosystems, as well as to national economies, through the introduction of agricultural and forestry pest species or by causing public health problems (e.g. transportation of malaria or dengue mosquitoes). Cruise ships are particularly susceptible to invasion by non-indigenous species, with large numbers of people from geographically diverse areas providing a regular supply of non-native propagules^5^. According to the pathways described by Hulme *et al*.^2^, cruise vessels may act as vectors for terrestrial NIS arthropods via the following routes: (1) contamination (e.g. in food sources^17^), ornamental plants, wood, animals, fresh water storage), (2) stowaways - accidental association with any cargo on the vessel, or (3) corridors - transport infrastructure linking previously unconnected regions. The Australian Government considers cruise vessels a high biosecurity risk due to the large number of passengers disembarking with souvenirs from other countries potentially containing items of biosecurity concern, the considerable amount of food and stores on board, waste management and the possibility that live plants are kept on the vessel (http://www.agriculture.gov.au/biosecurity/avm/vessels/commercial-vessels#cruise-vessels). In recognition of the biosecurity risks posed by vessels, the New Zealand government has biosecurity procedures in place for arriving vessels^18,19^ and has recently launched a biosecurity trial targeted at the food passengers take from the cruise ships when disembarking (http://www.mpi.govt.nz/news-and-resources/media-releases/cruise-ship-biosecurity-trials-to-begin/).

The risk of NIS establishment via human-mediated pathways increases every year as travel and global trade grows^20,21^. Cruise tourism is the fastest growing segment of the global tourism industry^22^, estimated to grow at a compound annual growth rate of 6.6% until 2020 (http://www.cruisemarketwatch.com/growth/). Since 2007 ocean cruise passenger numbers in Australia have had an average annual increase of 19.4%, with a record of 1.2 million passengers in 2016. By 2020, the industry’s target is for two million passengers^23^. Coupled with this, cruise ships are making more frequent visits to ports that previously experienced limited vessel traffic. Offshore anchoring and transport of passengers to remote areas is also becoming more common^24^.

The passenger vessel under consideration in this study had been in service since 1993. In 2014 the vessel began a 31-day trip from Estonia, travelling to Barrow Island, located off the north west Australian coastline. Upon arrival, the vessel was stationed in the island’s waters for a 15-month period. The vessel was used as a static accommodation facility to house workers employed as part a liquid gas project (Gorgon Gas Development) in operation on the island. The biosecurity surveillance and eradication program were conducted during the voyage to Australia and while the vessel was berthed at Barrow Island. A condition of the gas development approval was the inclusion of a NIS detection program that could detect if NIS arrived on the island due to Gorgon Gas Development activities^25,26^. A detailed post-border surveillance program has been implemented on Barrow Island to ensure early detection of NIS. This surveillance program has been ongoing since 2009.

This paper provides an account of the first known biosecurity management programme that has attempted to eradicate terrestrial arthropods on a large passenger vessel. The aim of this biosecurity measure was to prevent the introduction of NIS to Australia. We provide details relating to this eradication attempt. This study also documents the longest and most extensive assessment of the range of terrestrial arthropods that can persist on a large passenger vessel, with the vessel in service since 1993. The study provides details showing that different areas of large vessels can support distinct arthropod assemblages. Given the high biosecurity risks posed by these passenger vessels and the continued rise in vessel movement, there is clearly a need for a better understanding of the composition of terrestrial arthropods that may be present on them and effective strategies for managing and eradicating these hitchhikers.

## Results

### Arthropod detections pre-departure from estonia

After completing an on-board risk assessment and vessel refurbishment prior to leaving Estonia, the following nine species were detected on board: *Dermestes* sp., *Dermestes lardarius* Linnaeus, *Drosophila repleta* Wollaston, *Liposcelis sp*, *Staphylinidae* sp., *Formicidae* sp., *Oryzaephilus mercator* (Fauvel), *Attagenus smirnovi* Zhantiev and *Willowsia buski* (Lubbock). Of these, only one species was identified as a species of concern; *D*. *repleta* (ferment fly).

### Arthropod detections during voyage from estonia to dampier, Australia

Of the nine species detected pre-departure from Estonia, three species continued to be found during the voyage from Estonia to Dampier, Australia: *D*. *repleta*, *A*. *smirnovi* and *O*. *mercator*. One additional NIS species was detected during this voyage period (*Drosophila melanogaster* Meigen 1830), and another NIS species was detected when the vessel was in Australian waters but had not yet entered port (*Tribolium destructor*). Two of these species, *T*. *destructor* and *A*. *smirnovi*) are considered NIS to Australia, with *T*. *destructor* of greatest concern due to its potential impact on Australia’s grain industry. All five species are NIS to Barrow Island (BWI).

### Arthropod detections and eradication at barrow island, Australia

During the 15 months that the *Europa* was berthed at Barrow Island, 17,608 personnel screenings were conducted as personnel departed the vessel for the mainland, or transferred to other accommodation facilities on Barrow Island. No NIS were detected during this screening.

**Figure 1:**
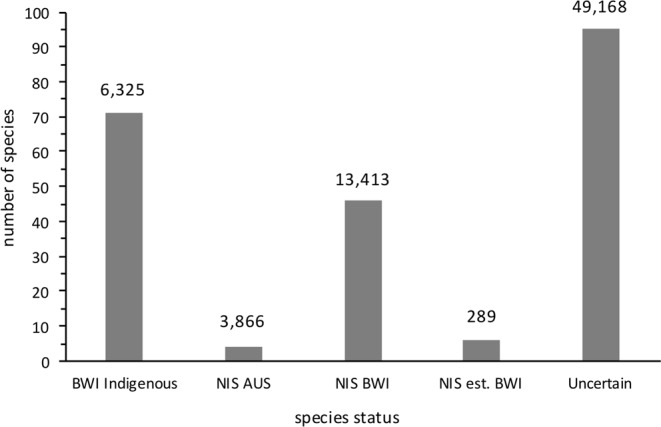
Number and abundance of species collected from the *Europa* according to arthropod status. Numbers above each column show the total number of arthropods collected for that status group. BWI Indigenous = species is indigenous to Barrow Island (BWI); NIS BWI = species not previously detected on Barrow Island; NIS est. BWI = NIS already established on Barrow Island, NIS Aus = species is NIS to Australia; Uncertain = uncertain species identification or status.

While berthed at Barrow Island, 73,061 arthropod specimens (222 species) were collected from the *Europa*. This included specimens from the nine species detected pre-departure. Of the 73,061 specimens, 67.3% (49,168 specimens; 95 morphospecies) were unable to be identified to the lowest taxonomic level (see methods), and were consequently assigned the status of ‘uncertain’ (Fig. 1). These specimens are not considered further in this section of the results.

The remaining 23,893 specimens were from 127 species. Of these, 26.5% had previously been collected from Barrow Island as part of the NIS surveillance program and were indigenous to Barrow Island (6,325 specimens; 71 species). A further 1.2% of species had been previously collected from Barrow Island and were non-indigenous species that were already established on Barrow Island prior to the Gorgon project commencing in 2009 (289 specimens; 6 species). The remaining 72.3% of specimens collected (17,279 specimens; 50 species) had not been previously collected from Barrow Island. Four of these species were confirmed as non-indigenous to Barrow Island (3,866 specimens), Western Australia or Australia (*T*. *destructor*, *A*. *smirnovi*, *D*. *filum*, *Anthocomus rufus* (Herbst)).

**Figure 2:**
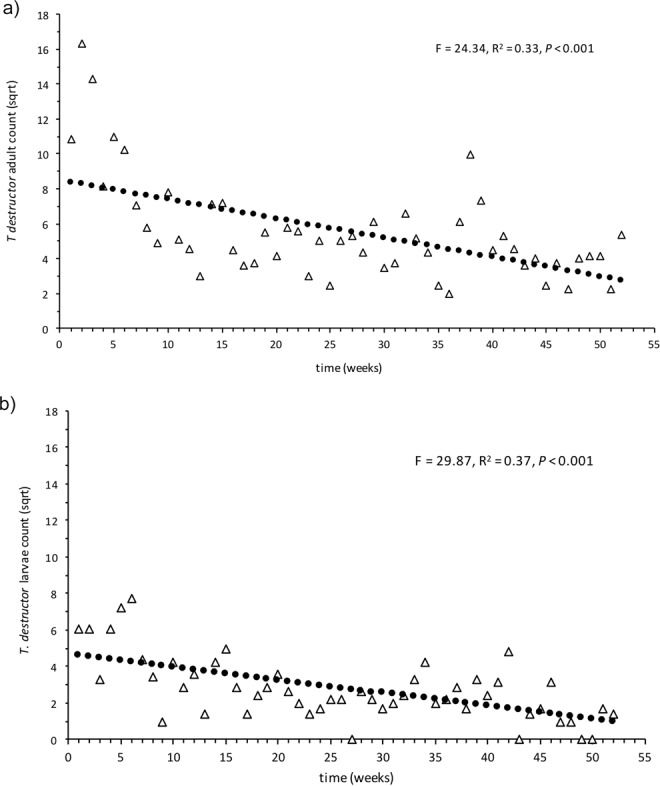
Regression plot of total weekly counts for *Tribolium destructor* on decks 7 and 8 for the period of vessel surveillance. Data were square root transformed. (**a**) Adults: R^2^ = 0.33, *P* < 0.001, df = 50, (**b**) larvae: R^2^ = 0.37, *P* < 0.001, df = 50.

Of the four NIS to Australia species identified, *T*. *destructor* was of greatest concern to Australian Government Department of Agriculture and Water Resources as it was assessed as posing a significant risk to the Australian grains industry. It was not, however, considered a risk to Barrow Island biodiversity (http://www.agriculture.gov.au/SiteCollectionDocuments/ba/memos/1999/plant/TWGP_1.pdf). *T*. *destructor* was the sixth most commonly collected species, most likely due to targeted surveillance for this taxon (outlined in methods) (2601 individuals, Table 1). This species was detected on three decks; 6, 7 and 8 (Table 1). All three of these decks were food processing and consumption areas. *T*. *destructor* was never detected in the cabins. There was considerable overlap in areas where *T*. *destructor* was found when the three decks were overlayed. No evidence could be found to indicate that the beetles were moving from one deck to another but common usage of these areas for food and drink consumption suggests that the provision of a food source in these areas was highly suitable to recruitment of this species. Collection of *T*. *destructor* on Decks 7 and 8 decreased significantly over the time the vessel was berthed at Barrow Island (adults: df = 50, R^2^ = 0.33, F = 24.34, *P* < 0.001; and larvae: df = 50, R^2^ = 0.37, F = 29.87, *P* < 0.001; Fig. 2a,b respectively).

**Table 1:** Non-indigenous arthropods identified on the Europa for the period the vessel was at Barrow Island (surveillance period 19 November 2014 – 27 November 2015).

Species	Number of individuals: adult (larvae)	Number of decks detected on	Deck of collection
**NIS Australia**			
**Insecta - Coleoptera**			
Tribolium destructor (Destructive flour beetle)	2096(505)	3	6,7,8,NR
Dienerella filum (Minute mould beetle)	1 247	3	2,7,8
Attagenus smirnovi (Vodka beetle)	7(10)	6	5,7,8,9,11,13,NR
Anthocomus rufus (Soft-winged flower beetle)	1	1	7
**Most abundant NIS BWI**			
**Arachnida - Acari**			
Acarus siro (Stored product mite)	244	2	7,8
Blattiscoiidae sp.1 (Mite)	10	1	8
**Entognatha - Collembola**			
Willowsia buski (Springtail)	13	1	1
Insecta - Coleoptera			
Ahasverus advena (Foreign grain beetle)	346(37)	2	7, 8
Attagenus sp. (Beetle)	43	8	1,3,4,6,7,8,10,13
Oryzaephilus mercator (Merchant grain beetle)	1 991	3	1,7,8
Reesa vespulae (Carpet beetle)	283(2436)	6	3,6,7,8,9,12
Sitophilus oryzae (Rice weevil)	614(8)	1	1
Stegobium paniceum (Drugstore beetle)	65	1	1
**Insecta - Diptera**			
Drosophila melanogaster (Vinegar fly)	68	8	1,23,6,7,8,10,12,NR
Drosophila repleta (Vinegar fly)	500(51)	10	1,2,3,5,6,7,8,10,11,12
Drosophilidae sp. (Fly)	40	1	7
Lucilia cuprina (Blowfly)	10	1	NR
Megaselia scalaris (Scuttle fly)	6 331	13	All decks
Megaselia sp. 1 (Scuttle fly)	52	3	1,3,8
Musca domestica (House fly)	11	2	1,3
Mycetophilidae sp.(Fungus gnat)	11	2	1,3
**Insecta - Psocoptera**			
Lepinotus patruelis (Booklouse)	17	2	1,7
Lepinotus sp (Booklouse)	184	2	7,8
**Insecta - Zygentoma**			
Lepisma saccharina (Silverfish)	34	2	7,8
**NIS established BWI**			
**Insecta - Blattodea**			
Supella longipalpa (Brownbanded cockroach)	1	1	3
**Insecta - Coleoptera**			
Lasioderma serricorne (Cigarette beetle)	150(3)	3	1,7,8
**Insecta - Psocoptera**			
Dorypteryx domestica (Psocid)	49	1	8
Liposcelis bostrychophila (Booklouse)	48	4	3,4,7,8
Liposcelis entomophila (Booklouse)	2		8
**Insecta - Zygentoma**			
Ctenolepisma longicaudata (Long-tailed silverfish)	36	4	5,6,7,8

NIS Australia = species NIS to Australia, NIS BWI = species not previously detected on Barrow Island (the most abundant listed); NIS established BWI = NIS already established on Barrow Island; NR = deck not recorded. Numbers in parentheses represent larvae.

**Figure 3:**
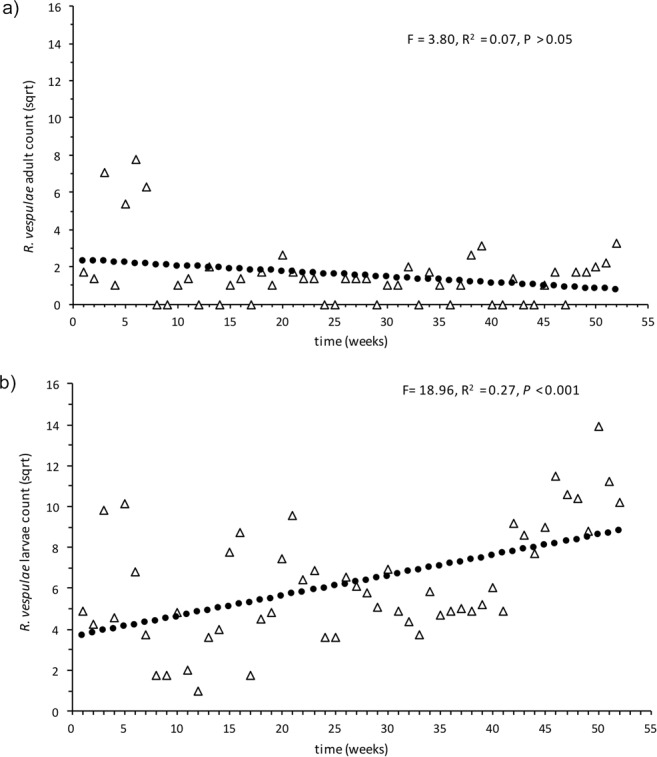
Regression plot of total weekly counts for *Reese vespulae* on decks 7 and 8 for the period of vessel surveillance. Data was square root transformed. (**a**) Adults: R^2^ = 0.07, *P* = 0.057, df = 50, (**b**) larvae: R^2^ = 0.27, *P* < 0.001, df = 50.

**Figure 4:**
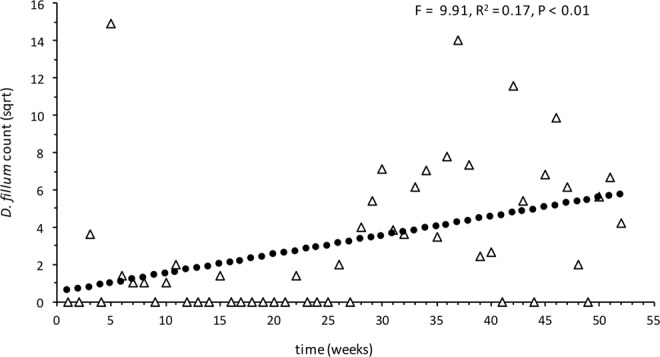
Regression plot of total weekly counts for *Dienerella filum* on decks 7 and 8 for the period of vessel surveillance. Data was square root transformed. R^2^ = 0.17, *P* =  < 0.01, df = 50.

The eradication effort incidentally targeted the other beetle species that were considered non-indigenous (*Attagenus smirnovi*, *Dienerella filum*, *Anthocomus rufus* and *Reesa vespulae*) (Table 1). As the niche for each of the beetle species was found to be very similar, a single methodology for eradication was undertaken. In total, 1,247 (adult and larvae) *D*. *filum*, 2,719 (adult and larvae) *R*. *vespulae*, and 17 adult (no larvae) *A*. *smirnovi* were detected. Like *T*. *destructor*, *R*. *vespulae* was restricted to a limited number of decks (see Table 1), with the majority detected on Decks 7 and 8. For *R*. *vespulae*, there was no significant change in adult numbers over time (adults: df = 50, R^2^ = 0.07, F = 3.80, *P* = > 0.05, Fig. 3a). In contrast, *R*. *vespulae* larvae numbers increased significantly over time (df = 50, R^2^ = 0.27, F = 18.96, *P* < 0.001, Fig. 3b). *D*. *filum* was found on decks 1, 3, 7, and 8 (Table 1), with the majority detected on Decks 7 and 8. The numbers of adult *D*. *filum* increased significantly over time (df = 50, R^2^ = 0.17, F = 9.91, *P* < 0.01, Fig. 4). No larvae of this species were detected (Table 1).

At the completion of the 15-month surveillance period on Barrow Island (30 December 2015), *T*. *destructor*, *D*. *filum* and *R*. *vespulae* were still being detected on the vessel. *A*. *smirnovi* was last detected on 18 June 2015. All detections were made via visual inspections with no target beetles detected in lure traps.

All nine NIS species detected prior to departure from Estonia as part of the on-board risk assessment (see above) were collected over the 15-month surveillance period while the vessel was berthed at Barrow Island (Table 1). However, none of the NIS detected on board the *Europa* have been subsequently detected on BWI as part of the NIS surveillance program in place under the Gorgon Gas Development.

### Arthropod hot-spot locations on the vessel and arthropod assemblage structure

There was a significant difference in the assemblage of arthropods found between decks (ANOSIM: R = 0.31, *P* = 0.001). Deck 1 (food preparation and storage) had a significantly different terrestrial arthropod assemblage from all other decks on the vessel, as did Decks 7 and 8 (food and alcohol consumption areas) (Supplementary Fig. S1). The array of Diptera, Coleoptera, Sarcoptiformes and Psocoptera contributed to the differences in arthropod assemblage composition (Supplementary Table S1).

**Figure 5:**
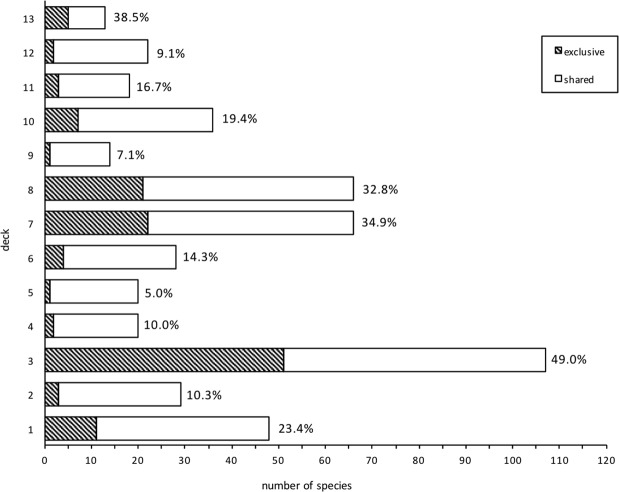
Total number of species collected from each deck of the vessel, showing the breakdown of species found exclusively on each deck and those shared with other decks. Numbers beside columns represent the percentage of species exclusive to that deck.

Of the 222 species collected during the 15 months the Europa was berthed at Barrow Island, 54.1% were collected on only one occasion and consequently many were exclusive to the deck on which they were collected (Fig. 5). There were, however, ten species that were highly abundant (Table 1), and represented 89.7% of all arthropods collected. These species comprised of Diptera (6 species) and Coleoptera (4 species) (Table 1). The two Diptera species were *Megaselia scalaris* (NIS established to Barrow Island - found on all decks of the vessel) and *Musca vetustissima* (indigenous to Barrow Island). All four highly abundant Coleoptera species were either NIS to Australia or had not previously been detected on BWI (Supplementary Table S1). Overall, Diptera species comprised 81.7% of all arthropods collected, with Coleoptera the next most abundant order (14.6%) (Table 2).

**Table 2:** Contribution of various arthropod orders to the total number collected over the entire vessel.

Arthropod order	Contribution in terms of number of individuals (%)
Diptera	81.7
Coleoptera	14.6
Sarcoptiformes	1.8
Psocoptera	0.6
Lepidoptera	0.4
Hemiptera	0.4
Thysanura	0.1
Other *	0.3

*includes, by order of abundance Acarina, Hymenoptera, Isoptera, Neuroptera, Mesostigmata, Entomobryomorpha, Araneae, Blattodea, Odonata, Ixodida, Collembola, Julida, Dermaptera.

There were specific areas on the *Europa* that were hotspots for arthropods; 72.3% of the total specimens (52,798 individuals; 104 species) were collected from Deck 3, an open access deck allowing vehicle and personnel admittance (Fig. 5, Supplementary Table S1). Of the 54 species that could be identified from Deck 3, 40 species (74.1%) were indigenous to Barrow Island and 4 species (7.4%) were NIS already established on the island. Ten species (18.5%) on this deck were species that had not previously been detected on BWI. Decks 8, 1 and 7 contained the next highest abundance of arthropods respectively (Supplementary Table S1).

## Discussion

In total, surveillance on board the *Europa* yielded 73,061 terrestrial arthropod specimens from 222 species. Of these, 50 species (17,279 specimens) had not been previously collected from Barrow Island. The number and range of species detected in this study highlight the potential biosecurity threat posed by cruise vessels. This is particularly pertinent given that an immense effort went into preparing the vessel for this work prior to departure and the ongoing surveillance on board the *Europa*. Most of the NIS collected on the *Europa* can be considered either synanthropic (e.g. German cockroach), or stored product pests (e.g. *Acarus siro* – stored product mite) and are unlikely to have an impact on the biodiversity of Barrow Island. A number of these species could, however, have posed a significant risk to the agricultural industry and the Australian national economy.

The very real threat to industry and the economy from these vessels is increased by the volume of cruise vessel traffic to Australian ports. For example, during the 2017/2018 cruising season, 352 cruise vessels visited Sydney alone, one of over 40 possible cruise stops in Australia (https://www.portauthoritynsw.com.au/cruise/cruise-industry/). As cruise tourism is the fastest growing sector of the global tourism industry^22^, considerable effort needs to be placed in biosecurity surveillance in this area. This study illustrates, however, that if stringent biosecurity protocols, combined with extensive eradication efforts, are put in place, the risk posed by cruise vessels can be reduced.

*Tribolium destructor* (destructive flour beetle) was one of the four species detected that were NIS to Australia. This species is known to cause significant damage to cereal products in many countries^27^ and has the potential to pose a significant risk to Australia’s grain industry if introduced to mainland Australia. Our study further highlights the risk posed by these NIS; the extensive eradication attempt on board the *Europa*, involving prolonged and intensive use of insecticides, baiting and trapping, did not fully eradicate *T*. *destructor* from the vessel. Indeed, there is no literature available of any successful eradication of terrestrial arthropods from a large vessel. Eradication efforts in general are rarely published^28^, representing an important gap in our current knowledge. This may reflect the limited success and the difficulty of the eradication task. The *Europa* was subject to the longest running and most extensive terrestrial pest monitoring programme recorded on a passenger vessel, occurring over a 19-month period, with 15-months of intensive surveillance and collection. The targeted eradication effort totalled more than 13,700 human hours and involved extensive baiting, trapping and chemical treatment on board the vessel. The eradication effort combined with the application of strict biosecurity protocols on board the vessel and Barrow Island facilities prevented the introduction of NIS to Barrow Island (see Scott *et al*.^29^) and Australia.

### Eradication successes and limitations

Most successful NIS eradications have concentrated efforts in the early phase of the invasion^30,31^. On vessels, successful NIS eradication may be constrained by late detection of the infestation. Arthropods that consume food material in storage, like *T*. *destructor*, may initially not be present in sufficient numbers to be noticed by crew members^32^. In the case of the *Europa*, the invasion of *T*. *destructor* likely occurred many years prior to detection. By the time the *Europa* arrived in Australia, the species of concern was well established in the many niches that existed on board, making eradication extremely difficult.

Another factor that may hinder eradication of terrestrial arthropods from vessels is the complexity of the vessel structure. On the *Europa*, despite evidence suggesting that *T*. *destructor* was restricted to just three decks, eradication of the beetle was not achieved. There were areas on the *Europa* where access for treatment was not possible. The extensive cleaning at the start of the eradication program at Barrow Island uncovered large volumes of potential arthropod food material, including a mix of human detritus, food crumbs and spilt drinks. This was despite the large clean and refit completed on the vessel in dry dock prior to departure from Estonia for Australia. The residual food was the accumulation of debris over the vessel’s 20 years of operation as a passenger ferry. It is very likely that any cruise vessel operating for more than a few years will also have a population of well-established arthropods in areas where food and drink is consumed. Indeed, Mouchtouri *et al*.^14^ reported older ferries were more heavily infested with cockroaches.

Although the eradication programme was unable to entirely remove *T*. *destructor* from the vessel, it was effective in controlling and decreasing their numbers. It was also successful in decreasing the abundance of adult *R*. *vespulae*. Interestingly, there was an increase in *R*. *vespulae* larvae abundance over time. It is unclear why this occurred, but it may indicate that food sources were diminishing, and larvae were becoming increasingly mobile in their search for food sources (see Hagstrum and Subramanyam^33^), or alternately that residual insecticide efficacy was beginning to subside. On the voyage from a cold Baltic Sea climate (average 9 °C maximum, Estonian Weather Service, https://www.ilmateenistus.ee/kliima/kliimanormid/ohutemperatuur/?lang=en) to Barrow Island, Australia (average 30 °C maximum, http://www.bom.gov.au/climate/averages/tables/cw_005058.shtml), the interior vessel temperature increased. The significant increase in *D*. *filum* numbers detected may be attributed to the subsequent increase in mould on the vessel as this species is mycophagous^34^. As the duration of the stay increased, humidity increased on the vessel due to the sub-tropical climate of Barrow Island. Subsequently higher mould levels were recorded. No larvae of *D*. *filum* were detected likely due to the fact that larvae of this family are very small^35^ and can remain hidden in breeding sites where females lay their eggs in the fungal food source e.g. in air-conditioning systems^36^ and under carpet, with beetles emerging from these sites in their adult phase.

Encouragingly, the extensive eradication efforts and strict biosecurity protocol implemented on the *Europa* prevented spread of NIS, with none detected leaving the vessel on passengers or their luggage and none have been detected during the subsequent island NIS surveillance program. Movement of personal luggage and waste from vessels poses the greatest risk of translocating pest species^14^, such as *T*. *destructor* and *R*. *vespulae*. In the case of the *Europa*, the volume of luggage leaving the vessel was small as most possessions remained on the vessel (in fit-for-purpose luggage rooms subject to monitoring and chemical treatment) between rotations (workers were rostered on 26-day rotations). In contrast, passengers on commercial cruise vessels will depart with large volumes of luggage regularly. Food waste from a vessel accommodating more than 1,000 passengers is estimated at 1 kg/passenger/day^37^ and without appropriate treatment the waste can pose a considerable biosecurity threat in introducing NIS to the surrounding systems. Freezing of food waste leaving vessels, as occurred on board the *Europa*, has the capacity to address this risk pathway. Furthermore, all clothing was washed and dried on board the *Europa* during the occupants’ stay. As part of the washing process, high temperature (60 °C) treatment was instigated on the *Europa* to reduce the likelihood of movement of arthropods off the vessel with clothing.

Visual surveillance was the best form of NIS detection during the eradication program. Even this, however, was hindered by the niches occupied by the organisms of concern. At the commencement of the eradication effort an extensive array of lures was distributed across the vessel to assist in determining the spatial extent of the NIS of concern. Unexpectedly, deployment of pheromone lure traps was not successful in attracting beetles. This was likely due to the broad application of other treatments, such as synthetic pyrethroids, used as part of the eradication attempt. It is also possible that establishment of an effective directional pheromone plume was inhibited because of the high number of lures set, coupled with the large volume of air exchange through the air conditioning system on board the vessel.

An advantage of eradication programs on vessels is that insecticides can be applied without the risk of collateral damage to non-target species. Fumigation with a chemical such as methyl bromide is normally an accepted treatment for any entity approaching a country’s biosecurity border. The Australian government states the standard treatment for invertebrates such as Khapra beetle, *Trogoderma graniarum* Everts is 80 g/m³ methyl bromide for 48 hours at 21 °C at normal atmospheric pressure, with an end point concentration at 48 hours of 20 g/m³ ^38^. This, however, is not an option for inhabited passenger vessels such as the *Europa*. The size of the vessel, the inability to isolate decks, uncertainty about ability to effectively vent after treatment due to adsorption of the chemical into carpets and upholstery, and the need to maintain crew on board negated fumigation as an option in this instance. Instead of broad scale fumigation, two groups of insecticides, with five different active ingredients, were utilised. These were sprayed in all possible areas where it was identified that populations of the target arthropods could persist, but again health and safety restrictions and inability to access all areas limited application. Health restrictions applied around food preparation and consumption areas. It was accepted that food stores had the potential to provide food and shelter for invasive species. As such, alternative biosecurity measures were implemented before the food was taken onto the vessel, strict hygiene requirements were maintained within the stores and there were regular inspections of stored products.

The health risk associated with spraying on board inhabited passenger vessels is not the only complication of eradication programmes. The presence of humans in areas of biosecurity concern is well known to complicate eradication, with successful eradication hinging on the engagement and support of inhabitants^39,40^. The *Europa* maintained a population of approximately 1,400 residents every day for the 15 months’ duration it was berthed at Barrow Island. Of these 1,400 residents, the vast majority co-operated with the eradication program. Furthermore, residents were encouraged to report any fauna (specifically arthropods) sightings to Chevron quarantine management. Adherence to biosecurity protocols and a strong biosecurity culture amongst personnel aided the eradication campaign^41^. Unauthorised consumption of food occurred in only a few cabins, but this was not considered to have impacted on the success of the eradication effort. Consequently, there was no evidence of spread of any of the target arthropods from the communal areas to cabins. To maintain co-operation, any safety concerns raised by the inhabitants in relation to the treatments, were addressed through communication, as recommended by Wilkinson and Priddel^42^.

### Arthropod hot-spots on the vessel

From the 15-months of surveillance conducted when the vessel was berthed at Barrow Island it was apparent that four decks on the vessel were hotspots for arthropod abundance: decks 1, 3, 7 and 8. Deck 3 was an open access deck to allow vehicles and personnel access to the vessel and thus it is not surprising that this deck showed the greatest number of detections (52,798 specimens). The multitude of the arthropods captured were mainly Diptera species that most likely flew onto the vessel while at the Barrow Island port and were caught in the light and sticky traps. In comparison, interceptions of pest insects at airports have been reported as largely comprised of Hemiptera, indicating that a distinctive suite of organisms is likely to be introduced by these different modes of transportation^43,44^.

Decks 1, 7 and 8 were also hotspots for arthropod detections, but for a different reason. These were areas where food and beverage were either stored or consumed, or where food had previously been consumed on the vessel prior to the refit. Consequently, there was an ample food supply for arthropods. Deck 1, the food storage deck, showed a significantly different arthropod assemblage to all other decks, largely due to the abundance of Diptera, Coleoptera and Hymenoptera species collected. It was apparent that the restaurants and bar on Decks 7 and 8, and areas previously used for food consumption, provided a plentiful food supply for arthropods despite the extensive cleaning programme that was undertaken. Both decks had a significantly different arthropod composition to the other decks (with the exception of Deck 6), likely reflecting the long-term access arthropods had to the food debris in these areas. These decks were hotspots for arthropod detections, with three of the four NIS to Australia species (*T*. *destructor*, *A*. *smirnovi*, and *D*. *filum*) inhibiting both of these decks. As such, these areas were targeted more intensively during the broad-scale eradication programme. The significant variation in arthropod abundance across the *Europa* should indicate to biosecurity managers more widely that it is advisable for initial surveillance to be broad-scale in approach, with more targeted surveillance in food consumption areas.

### Resource allocation for effective biosecurity

In order for biosecurity threats to be contained, more emphasis needs to be placed on screening potential vectors, such as large-vessels, that may carry contaminant and stowaway NIS. Screening will enable early warning of potential NIS and decisive action to be taken^45–47^. To succeed, however, there must also be access to sufficient resources. Regrettably this has hindered responses in many jurisdictions world-wide^47^.

The extensive eradication programme implemented on the *Europa* encompassed all decks of the vessel and is the most comprehensive and longest running monitoring and eradication programme recorded on any vessel. The outcome of the surveillance and eradication programme in this study are particularly pertinent given that Australian border inspection has previously been described as failing to detect a high percentage of exotic incursions into Australia^16^. This exemplifies the considerable biosecurity risk posed by cruise vessels, and other vessels more broadly, and the need for development of more effective screening and biosecurity programs, such as those implemented on board the *Europa*, to mediate the challenges created by these vessels.

## Materials and Methods

### Study site and biosecurity requirements

Barrow Island is situated off the north-west coast of Western Australia. It is a Class A Nature Reserve of high conservation value, containing many species that are now absent, or rarely seen, on mainland Australia^48,49^. A gas treatment facility that services the Gorgon and Jansz-Io gas fields (Gorgon LNG facility) is now in operation on Barrow Island. Preventing the introduction and establishment of NIS and invasive marine pests on Barrow Island was a key ministerial requirement of government approval for the Gorgon Project^29^.

To meet this ministerial requirement, the world’s most comprehensive biosecurity system was developed and implemented on Barrow Island; the Quarantine Management System (QMS)^29^. The objectives of the QMS is to prevent the introduction of NIS, and detect and eradicate NIS on Barrow Island and in its surrounding waters. In support of the QMS, a detailed baseline study of invertebrates was undertaken to determine if any NIS were already present on Barrow Island prior to commencing development of the Gorgon Project. Over 3430 terrestrial invertebrate species^41^ are now known from the island, a large proportion of which remain undescribed^50^. This baseline reference collection has proven invaluable in assessing a species’ status (non-indigenous or indigenous).

The QMS, also incorporates a vessel mobilisation procedure. In addition to vessel wetsides being free from secondary fouling, vessel topsides must be free from discernible evidence of soil, plants, plant material, seeds, invertebrates and vertebrates. To confirm the topsides status, it is a requisite for all vessels to undergo a 14-day baiting and trapping program for invertebrates and vertebrates, prior to being cleared for mobilisation to Barrow Island (see below for details). Prior to arriving at Barrow Island, it is mandated that all vessels undertake a 48-hour standoff, which requires no human activity on the vessels or sectors on a vessel in an approved sequence, during which additional invertebrate and vertebrate baiting is completed.

### Passenger vessel charter and description

In 2014, the Gorgon Project at Barrow Island required an increase in total workforce. As such, a large cruise ferry, the *Silja Europa* (*Europa*), was chartered. The *Europa* is one of the largest cruise ferries in the world and prior to the commencement of the charter it operated on the Tallinn-Helsinki route (Silja Europa Tallinn – https://www.tallinksilja.com/silja-europa-cruise-ship). The ferry, constructed in 1993, is 202 m in length, with a breadth of 32 m, with 1152 cabins and 13 decks (https://www.tallinksilja.com/silja-europa-cruise-ship). The vessel had operated solely in northern Europe from the time it was launched in 1993.

### Pre-charter survey and vessel refit in baltic sea

Prior to confirming the charter of the *Europa* in Estonia, a team of seven biosecurity specialists surveyed the vessel for high risk terrestrial pests. Pests included; rats (*Rattus rattus* Linnaeus 1758 and *R*. *norvegicus* Berkenhout, 1769), mice (*Mus musculus* Linnaeus 1758) and some invertebrate taxa (ants, cockroaches and spiders). The surveys consisted of approximately 500 hours of systematic visual inspections across accessible areas of the vessel, characterised by non-destructive examination of what were considered high risk niches (e.g. where food or drink was stored, prepared or consumed). Approximately 15% of accommodation areas were sampled, in conjunction with preliminary inspection of common areas. If animals, or signs of animals (e.g. scats) were detected, samples were collected and sent for identification by subject matter experts. This surveillance was undertaken while the vessel still contained a full complement of ferry passengers journeying between Finland and Estonia.

The vessel underwent a major refit when it was chartered (7 August 2014). The vessel was dry docked during the refit (24 August–7 September 2014) and all sea water systems and the hull were thoroughly cleaned. A new coat of antifoul was applied to reduce marine fouling. On board, all bedding and timber trim were replaced in cabins, kitchens and food storage areas. Most cabin carpets were replaced to mitigate possible arthropod risks. Flooring was replaced with linoleum in large common areas. Considerable effort was made to reduce arthropod food sources and available habitat. Refit and cleaning were completed in four weeks using 2000 workers. During the refit it was possible to inspect previously inaccessible areas. Considerable effort was made to mitigate risk of NIS incursion onto the vessel during the refit. This included wharf-based inspection and residual insecticide treatment of all new fittings prior to loading into the vessel, requiring another 2500 person hours. The functions of the main decks of the *Europa* are outlined in Table 3.

**Table 3:** Functions of the various decks on the *Europa* for the period it was berthed at Barrow Island.

Deck	Function
1	Food storage
2, 4, 5, 9, 10, 11, 12	Passenger cabins
3	Open car deck (vessel entry), passenger cabins
6	Crew cabins, crew dining area
7	Passenger dining area, shops, information area, gym: dining area)
8	Bar, internet café
13	Offices

### Pre-departure baiting and trapping program in baltic sea

Prior to sailing for Australia, the mandatory 14-day trapping and baiting program required for Barrow Island clearance was conducted on the *Europa* to ensure that the vessel was clear of any NIS. The baiting and trapping programme on-board included rat bait stations, installation of 210 UV light traps to remove/control the fly population, and more than 500 cockroach and ant trapping stations, in addition to treating the full exterior and most of the interior with residual insecticide (approximately 5000 litres at 1200 mL/100 L). Deltamethrin was selected as the insecticide’s active ingredient because of its strong residual characteristics and its broad-spectrum efficacy against invertebrates. Throughout preparation of the vessel, between two and four biosecurity specialists were present to oversee operations, continue visual surveillance for NIS and monitor the baiting and trapping programme.

Any species detected during this pre-departure phase was recorded and sent for identification at the Department of Primary Industries and Regional Development (DPIRD; previously Department of Agriculture and Food, Western Australia (DAFWA).

### Voyage from estonia to dampier, Australia

**Figure 6:**
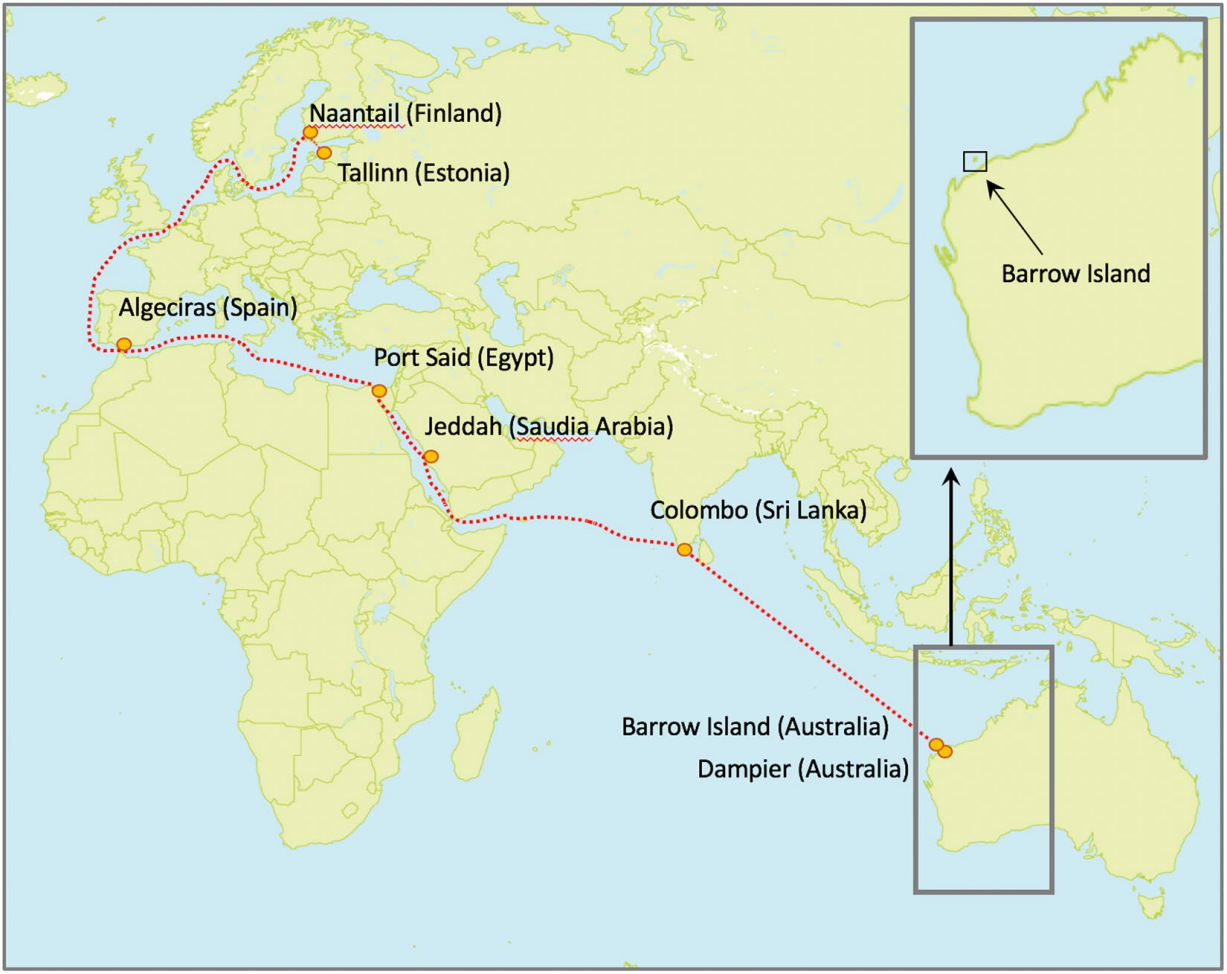
Route sailed by the *Europa* from Tallinn (Estonia) to Barrow Island (Australia). Ports (marked in red) indicate where the vessel stopped to bunker. Inset map shows location of Barrow Island off the west coast of Australia. Map created using a modified world map (WRLD-EPS-02–0012.jpg) sourced from FreeVectorMaps at http://freevectormaps.com.

The vessel departed Estonia for Australia on 12 September 2014 (see Fig. 6 for voyage course). Four Biosecurity Inspectors travelled with the vessel for the entire voyage. Throughout the 31-day trip they continued detailed visual inspection of the vessel. The trapping and baiting program (see above) continued throughout the voyage, including interior insecticide treatments of some areas. Part way through the voyage (between Port Said (Egypt) and Jeddah (Saudi Arabia) (Fig. 6), the vessel’s air-conditioning system failed, leading to an increase in heat and humidity on board the vessel. During every stop on the voyage from Estonia and Australia, anything leaving and coming onto the vessel was inspected by Quarantine personnel. These strict biosecurity measures were aimed at preventing the introduction of species to the vessel from the ports visited.

Between 28 September and 4 October 2014, prior to entering Australian waters, the vessel completed the Gorgon Project mandated 48-hour standoff period (see above). Rodent bait and trapping stations were monitored over 48 hours while the vessel was out of port. The vessel would only be cleared if there was no evidence of rodent activity for a 48 hour period.

On 13 October 2014 the *Europa* arrived in Australian waters and was required to anchor at Dampier (the nearest mainland port to Barrow Island) to complete Australian government customs and biosecurity requirements. An inspection of the vessel by Australian government authorities did not detect any organisms of concern and the vessel was approved for travel to Barrow Island. On 24 October 2014 the vessel berthed at Barrow Island and was occupied with personnel working on Barrow Island until its departure on 30 December 2015.

On the 17 October 2014, beetle specimens collected during the sail from Estonia to Dampier were identified by the DPIRD as *Tribolium destructor* (destructive flour beetle). This species is NIS to Australia. Following this identification, the Department of Agriculture and Water Resources (DAWR; previously known as the Australian Government Department of Agriculture (DoA)) placed the vessel under quarantine for its entire stay in Australia and a *T*. *destructor* management plan was devised.

### Management and attempted eradication of T. destructor

The management/eradication plan for *T*. *destructor* consisted of; i) an initial treatment phase prior to the vessel being occupied by personnel and, ii) an ongoing management/eradication campaign.

#### Initial treatment phase

The initial treatment phase (18 to 29 October 2014) involved 18 staff (six biosecurity inspectors, six general workers and six pest controllers) who worked across two shifts, 24 hours per day, to complete the required tasks. Tasks were: (1) cleaning, (2) chemical treatments and (3) delimiting. Cleaning included removal of any waste and debris associated with human and invertebrate activity by vacuuming and washing. Chemical treatment involved the application of a variety of insecticides (deltamethrin, bifenthrin, imidacloprid/beta-cyfluthrin or permethrin dust) in an attempt to reduce the instance of arthropods developing resistance to the chemicals. Insecticides were applied at approved rates to deconstructed areas. In addition to this, any loose seating that had significant wood material coverings was treated to ISPM 15 requirements. ISPM 15 is the international standards for phytosanitary measures for the treatment of wood materials of a thickness greater than 6 mm. Delimiting involved visually inspecting and partial deconstruction of areas suspected to be beetle niches e.g. lifting unreplaced carpet edges, removing metal flooring trim and undoing and lifting bench seat bases. Any insect activity was recorded. Delimiting surveys were structured such that each of the 13 decks were cleared (i.e. cleaned, treated and checked for beetle activity) in a sequential process.

#### Ongoing management/eradication campaign

**Figure 7:**
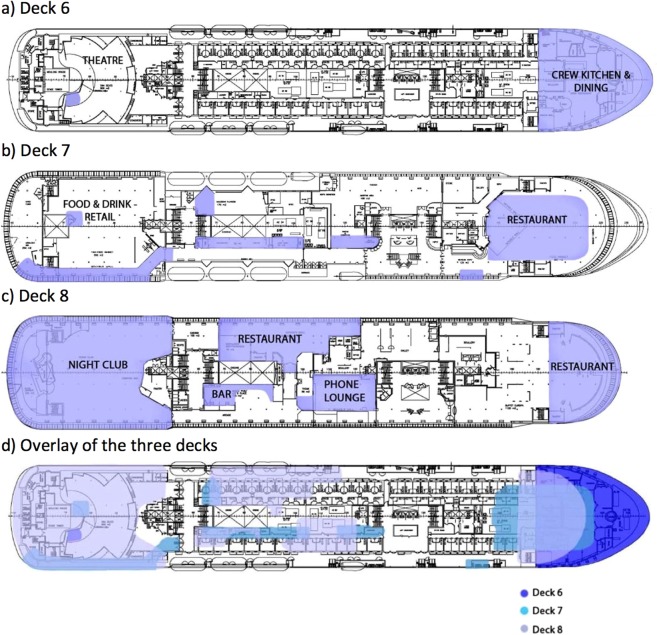
Deck maps indicating various functions on the vessel prior to its movement to Barrow Island and locations where *Tribolium destructor* was detected (**a**) Deck 6, (**b**) Deck 7, (**c**) Deck 8, and (**d**) overlay of Decks 6, 7 and 8. Shaded areas mark the general location of detections. The Food and Drink retail area on Deck 7 was converted to a gym and the night club area on Deck 8 was used as a bar during its time at Barrow Island.

The eradication phase commenced on 29 October 2014, taking more than 13 700 hours over a 15-month period (until 30 December 2015). The surveillance component of the eradication phase included both visual inspections and pheromone traps and lures. Surveillance components were deployed in identified risk areas as well as *ad hoc* locations throughout the vessel. The identified risk areas were based on the *T*. *destructor* delimiting surveillance and were areas where food or drink was stored, prepared or consumed (predominantly on Decks 6, 7 and 8) (Fig. 7). The number of arthropods collected were recorded on a weekly basis. Deck maps were used to maintain a record of where each sample was collected (Fig. 7).

All specimens that were collected were forwarded to DPIRD for species level identification. In some instances, species level identification was not possible. This occurred when the specimen was of a problematic life stage for species level diagnostics, or damaged. In these cases, the specimen was assigned a morphospecies name according to the lowest taxonomic level identified. Both species and morphospecies have been combined for data analysis and, unless specified, are termed ‘species’ for the purposes of this paper. The status of each species was assigned to one of the following: (1) indigenous to Barrow Island; 2) NIS previously established on Barrow Island; (3) NIS to Barrow Island; or (4) NIS to Australia. Any detection of a NIS to Australia was reported to DAWR within 24 hours.

Eighty-three pheromone lure traps utilising different pheromones as well as wheat germ bait, were deployed across all decks of the vessel and monitored weekly for a period of 3 months. Pheromones from the following species were utilised; *Tribolium* spp., *Sitophilus oryzae*, *Rhyzopertha dominica*, *Trogoderma variabile* (Insects Limited Inc). The lures were selected after a search for semiochemicals of *T*. *destructor* on Pherobase (http://www.pherobase.com/database/species/species-Tribolium-destructor.php). Lures were placed into insect trapping stations and adhesive paper was fitted to the bases. Traps were designed to lure and kill arthropods. Lures were replaced according to the manufacturer guidelines.

A scheduled insecticide treatment was implemented as part of the ongoing treatment plan. Insecticide was applied to different areas of the vessel on either a four or eight-week schedule depending on the risk assigned. High risk areas were those with higher human activity or were likely to contain food sources for the target species. High risk areas received insecticide applications every four weeks. Three different synthetic pyrethroids (bifenthrin, deltamethrin and beta-cyfluthrin) as well as a neo-nicotinoid (imidacloprid) were rotated to increase insecticide effectiveness compared to using a single insecticide.

All materials leaving the vessel were treated as required by DAWR as follows: all food waste was frozen at −18^2014–27^C for 7 days, while all other waste, used linen and other materials were fumigated with methyl bromide (48 g/m^3^ for 24hrs) before being approved by DAWR for removal to the mainland for disposal.

An enhanced biosecurity awareness campaign among vessel inhabitants was conducted. The campaign consisted of inductions to the vessel that included an introduction to the eradication effort, and distribution of flyers/posters throughout the vessel. The key objectives of the communications were to (1) stress the importance of the eradication effort in protecting the biodiversity of Barrow Island and Australia’s valuable grain industry, and (2) restrict the spread of beetles on the vessel from the common areas to the cabin decks by ensuring that inhabitants only consumed food in designated areas and did not bring any food onto the vessel that may provide a food source for the beetle. The campaign continued for the full term the vessel was present at Barrow Island. This type of campaign has proven highly effective amongst personnel on Barrow Island^41^.

It was quickly identified that prohibiting food consumption in the cabins was not achievable. As such, this position was relaxed to only prohibiting foodstuffs that were likely food sources for the targeted NIS (e.g. potato crisps, bread, biscuits or similar grain products). Inhabitants on the vessel complied with the food restriction requirements. Cleaning and insecticide treatment teams were vigilant and reported cabins where high risk foodstuffs were found. Occupants of such cabins received a letter informing them of the need to comply with the food consumption requirements of the vessel and additional insecticidal treatment was applied where appropriate.

All passengers departing the vessel were required to present their luggage for inspection. Biosecurity inspectors examined the exterior of all luggage, with a focus on niches where invertebrates may be present, including pockets, wheel recesses and zips. Random inspections of bag interiors (>5%) were undertaken by biosecurity inspectors, with personnel consent.

### Statistical analysis

Total number of arthropod specimens collected were tallied for each deck and location, and for the vessel, to determine the total number of arthropod species (including morphospecies) present and arthropod abundance. For three of the most common NIS detected (*T*. *destructor*, *Reesa vespulae* (Milliron), and *Dienerella filum* (Aubé)) abundance data were square-root transformed to ensure homogeneity of variance, and regression analysis were conducted to determine if there were any changes in abundance over the 15-month eradication period.

Differences in arthropod order composition (based on densities) across the vessel were investigated using a Bray-Curtis similarity index (data were square-root transformed prior to analysis), followed by an analysis of similarity (ANOSIM)^51^ to test the hypothesis of differences in arthropod order among decks. Probability levels were set at P < 0.05 for all tests.

## Supplementary information

Supplementary Information
