# Supplementary material for: Biosecurity risks posed by a large sea-going passenger vessel: challenges of terrestrial arthropod species detection and eradication
Source: Sci Rep. 2019 Dec 18;9:19339. doi: 10.1038/s41598-019-55554-4 (PMC6920439; doi:10.1038/s41598-019-55554-4)
Supplement: Supplementary file 1 — Supplementary Information [file 41598_2019_55554_MOESM1_ESM.docx]

**Supplementary Information**

**Biosecurity risks posed by a large sea-going passenger vessel: challenges of terrestrial arthropod species detection and eradication**

**Simon J. McKirdy ^1*^, Simon O’Connor^1,2^, Melissa L. Thomas^1,2^, Kristin L. Horton^2^, Angus Williams^2^, Darryl Hardie^3^, Grey T. Coupland^1^ and Johann van der Merwe^1,2^**

* corresponding author: [s.mckirdy@murdoch.edu.au](mailto:s.mckirdy@murdoch.edu.au), tel: +61 8 9360 2062

^1^Harry Butler Institute, Murdoch University, Murdoch WA 6150, Australia

^2^Chevron Australia, 256 St Georges Tce, Perth WA 6000, Australia.

^3^Department of Primary Industries and Regional Development Western Australia, Division of Agriculture and Food, 4 Baron-Hay Court, South Perth 6151

| 2 | **−** |  |  |  |  |  |  |  |  |  |  |  |  |
| --- | --- | --- | --- | --- | --- | --- | --- | --- | --- | --- | --- | --- | --- |
| 3 | **−** | **+** |  |  |  |  |  |  |  |  |  |  |  |
| 4 | **−** | **+** | **+** |  |  |  |  |  |  |  |  |  |  |
| 5 | **−** | **+** | **+** | **+** |  |  |  |  |  |  |  |  |  |
| 6 | **−** | **+** | **+** | **+** | **−** |  |  |  |  |  |  |  |  |
| 7 | **−** | **−** | **−** | **−** | **−** | **+** |  |  |  |  |  |  |  |
| 8 | **−** | **−** | **−** | **−** | **−** | **+** | **+** |  |  |  |  |  |  |
| 9 | **−** | **+** | **+** | **+** | **+** | **−** | **−** | **−** |  |  |  |  |  |
| 10 | **−** | **−** | **+** | **+** | **+** | **+** | **−** | **−** | **+** |  |  |  |  |
| 11 | **−** | **−** | **+** | **+** | **+** | **−** | **−** | **−** | **+** | **+** |  |  |  |
| 12 | **−** | **−** | **+** | **+** | **+** | **+** | **−** | **−** | **+** | **+** | **+** |  |  |
| 13 | **−** | **−** | **+** | **+** | **+** | **+** | **−** | **−** | **+** | **+** | **+** | **+** |  |
|  | 1 | 2 | 3 | 4 | 5 | 6 | 7 | 8 | 9 | 10 | 11 | 12 |  |

**Supplementary Figure S1.** Similarity matrix of ANOSIM results for decks on the *Europa*. ‘+’ = no significant difference in arthropod assemblage composition between decks, ‘−’ significant difference between decks in arthropod assemblage composition

**Supplementary Table S1.** Abundance of various arthropod orders collected from each deck of the vessel. NR = not recorded
